# Dose prediction for cervical cancer VMAT patients with a full-scale 3D-cGAN-based model and the comparison of different input data on the prediction results

**DOI:** 10.1186/s13014-022-02155-7

**Published:** 2022-11-13

**Authors:** Gongsen Zhang, Zejun Jiang, Jian Zhu, Linlin Wang

**Affiliations:** 1 Artificial Intelligence Laboratory, Shandong Cancer Hospital and Institute, Shandong First Medical University, Shandong Academy of Medical Sciences, Jinan, Shandong China; 2 Department of Radiation Oncology, Shandong Cancer Hospital and Institute, Shandong First Medical University, Shandong Academy of Medical Sciences, No. 440, Jiyan Road, Huaiyin District, Jinan, Shandong China; 3 Department of Radiation Oncology Physics and Technology, Shandong Cancer Hospital and Institute, Shandong First Medical University, Shandong Academy of Medical Sciences, Jinan, China

## Introduction

Cervical cancer is the second most common female malignant tumor in the world [1], for which radiotherapy is currently one of the main treatment methods. Related surveys show that approximately 80% of cervical cancer patients receive radiotherapy at different stages [2, 3]. Intensity modulated radiotherapy (IMRT) and volumetric modulated arc radiotherapy (VMAT) have become standard radiotherapy methods. Compared with 3-dimensional conformal radiotherapy (3D-CRT), the dose distribution formed by new technologies above using reverse optimization algorithms is highly consistent with the planned target area and has better uniformity[4–7]. However, advanced technology also brings corresponding computational burden, which greatly increases the total planning time. According to statistics, it takes an average of approximately 4 h for radiotherapists to delineate the planning target volume (PTV) and organs at risk (OARs), and may even take longer for some complex diseases. After that, a radiotherapy plan meeting the treatment standards is formulated by radiation physicists, which takes approximately 10 h for each patient [8, 9]. The large amount of time required for the treatment plan inevitably leads to delayed treatment, thereby affecting the quality of treatment and prognosis of patients [10].

In order to address the disadvantages mentioned above, automatic planning and dose distribution prediction for radiotherapy planning has been widely considered in recent years. The relevant research is mainly carried out through knowledge-based projects (KBP) [11–15]. Some studies help physicists in planning designs by enumerating dose parameters and characteristics into the DVH model [16–19]. However, manual intervention is inevitable in these methods, on the other hand, the dose distribution of the undepicted tissue structure is ignored. With the advancement of hardware technology, deep learning represented by convolutional neural networks has become increasingly popular. In the research on computer vision and medical image processing, the advantages of using the network architecture for deep learning are adopted to extract deep feature information to analyze and process data, which simplify calculation procedures and obtains a reliable accuracy to a large extent [20–24]. In the field of radiotherapy, Dan Nguyen et al. used a U-net convolutional neural network to predict dose distribution. They use the outline of the planned target area and the OARs as input to establish a correlation between the outline and the dose value [25]. Similar studies have also proven the potential of deep learning in dose prediction [26–31].

Although the existing prediction models based on deep learning have achieved considerable consistency between the clinical and predicted dose distribution, these studies still have some shortcomings. First, the current deep learning-based dose prediction research mainly uses the mean square error (MSE) between the predicted dose and the clinical dose as a loss function to perform gradient descent, and finally find the best solution. The MSE loss function adapts to the uncertainty in the prediction by taking the average of the possible outputs. It is a fuzzy prediction method that reduces the detailed information of the image. Second, most of the existing research model structures and model inputs are implemented in two dimensions, However, the dose distribution of the radiotherapy plan is related not only to the current slice, but also to the adjacent slices. With a two-dimensional model, key information may be discarded. Third, these studies still require clinicians to delineate OARs, which is a rather time-consuming process. If the dose distribution can be predicted without delineating the OARs, considerable treatment planning time would be saved, which would allow clinical radiotherapists and physicists to focus their time on more challenging situations or demanding tasks.

Specifically for image processing related tasks, Ian Goodfellow et al. proposed a generative adversarial network (GAN), which is a new type of unsupervised architecture consisting of two independent networks, a generation network (generator) and a discriminating network (discriminator) [32]. Based on the idea of GAN, Mirza et al. proposed the conditional generative adversarial networks (cGAN), in which specific condition information is added, taking the place of random noise, to realize supervised learning during the image generation process [33]. The generator is used to generate an image, which is judged as a real image after passing through the discriminator, while the discriminator is used to estimate the probability that the current sample belongs to the real image. This leads to a process of mutual confrontation, in which the generator tries to generate synthetic images similar to the real images to confuse the discriminator, and make it classify the result as “real”, while the ideal training result of the discriminator is to maximize the distinction between real and synthetic images, recognizing the result of the generator as “fake”. The performance of the two is improved in this mutual confrontation. When a “Nash equilibrium’’ point is reached, the image synthesized by the generator is considered to be a real image. Compared with the traditional convolutional neural networks, the parameter update of the generator comes from the backpropagation of the discriminator, rather than directly from the MSE. Mahmood et al. used the concept of style transfer to achieve pixel-level dose prediction based on GAN network with two-dimensional contoured computed tomography (CT) slices as input data [34]. Babier et al. realized dose prediction based on 3D GAN on the basis of traditional KBP method, and achieved better results than the 2D model [35]. These studies have explored the feasibility of GAN networks with a self-enhancing generative-adversarial learning architecture in an image generation task of dose prediction.

In this study, we propose a full-scale feature fusion 3D-cGAN-based deep learning network for 3D voxel-by-voxel prediction of the dose distribution in cervical cancer radiotherapy planning. There are many innovations in this work. First, we innovatively combined the idea of generative adversarial training strategy and 3D full-scale feature information [36], wherefore the input data information was fully utilized, and the architecture performance was enhanced during the mutual confrontation process between the two independent networks, to improve the accuracy of dose prediction; Second, we used a combination of multiple losses including the adversarial loss term, adjacent voxel dose difference loss term and L1 loss term function as the optimization target, instead of just using the MSE, to keep the edge features by minimizing the adjacent voxel dose value difference. In the process of model training, this restricted the difference in dose value changes between adjacent elements of the matrix, especially near the boundary of a specific area, while L1 paid more attention to the prediction dose matrix as a whole; Third, we tried to simplify the multichannel input data for dose prediction, but taking account of the clinicians’ experience and the individualized differences in patients with respect to the PTV [37, 38], which could not be completely replaced by CT anatomy information, we removed the OARs from the input data and established a dose prediction model that used only CT images and the PTV as input to address the time-consuming process of the clinical delineation of OARs. This proposed general method can realize the full potential of the deep neural network in dose distribution prediction, and provide a simpler and more effective method for clinical dose evaluation, radiotherapy planning assistance, as well as automatic planning.

## Materials and methods

### Patient database

The database collected in this research contained 118 cervical cancer patients treated with VMAT technology from 2017 to 2020 in Shandong Cancer Hospital. The studies involving human participants were reviewed and approved by Ethics Committee of Shandong First Medical University Affiliated Cancer Hospital (Approval ID: SDTHEC2020008005). Written informed consent for participation was not required for this study in accordance with the national legislation and the institutional requirements. The study was conducted in accordance with the principles of the Declaration of Helsinki and in accordance with local statutory requirements. All the raw data were exported from the Varian Eclipse Treatment Planning System (TPS) (Varian Medical Systems, Palo Alto, CA, USA), which included CT data, delineated contour information and clinical dose distribution map of each patient. The CT slices were obtained via scanning by a Siemens SOMATOM Confidence CT simulation positioning machine (Siemens Healthcare, Forchheim, Germany) while the patient was immobilized on a vacuum cushion. The PTV and OARs including the bladder, rectum and femoral heads on both sides, which revealed delineation information, were manually established and reviewed by experienced radiotherapists. The involved clinical dose distribution maps were acquired by iterative optimization according to empirical weight parameters and the radiotherapy plans based on that were approved by senior radiation physicists. The selected cases were all from the same group of radiotherapists, and they used the same delineation guidelines of NRG Oncology/RTOG [39–41]. The beam energy was 6 MV and all plans had been delivered and calculated through heterogeneity correction. The prescribed dose for all patients was 54 Gy, using a grading scheme: 180 cGy * 30 fractions. All the plans involved were formulated through three coplanar arcs, and no discrepancies existed in the clinical protocols and treatment criterion for any case.

### Dataset preparation and classification

The voxel value in 3D CT images was truncated, easily for standardization and normalization, while preserving valuable information, in the range of − 600 ~ 1000 HU (Hounsfield unit). The interval contained effective information since the pelvic cavity was the research object, a value below − 600 HU was basically air, and the bone information was included. With regard to contour data, masks of the body, femur-head-R, femur-head-L, rectum, bladder, and PTV were assigned various label values of − 400, − 200, 200, 600, 800, and 1000, respectively, which independently existed as binary images with the value of blank area set to -600. The pixel value of the RT Dose data exported from the TPS was converted to the dose value of the voxel through the dose grid scale factor. Taking into account the voxel-to-voxel level based on the establishment of correlation mapping relations, 3D input images mentioned above were all resampled to a space size of 3.5*3.5*3.5 mm^3^, assuring that the voxels contained the same physical-size information. In addition, all 3D data was cropped to matrix with a size of 128*128*128, considering the burden of model training as well as the preservation of the voxels which could be plenty utilized.

For all patients, 20 cases were randomly selected as the testing set, and the remaining 98 cases were divided into training-validation in a ratio of 6:1, to implement seven fold cross-validation. In order to cater to the original intention of this research, three datasets were formulated through different input data combination schemes, as shown in Table 1. For each dataset, the clinical dose distribution map was set as the first channel, which was used to compute the loss items and train the discriminator. In addition, Dataset-A was composed of data with 8 channels, in which channels 2–8 contained binary masks for the PTV, body and OARs delineation information; Dataset-C only contained two channels of which the second channel was CT images; Dataset-B includes 3 channels based on Dataset-C, where the PTV delineation information was added as a third channel to take into account the radiotherapist’s clinical experience and individualized differences in patients.

**Table 1:** Data composition of four multi-channel datasets

Datasets	Stacked data composition for generator
Dataset-A	Dose + CT + PTV + OARs + Body
Dataset-B	Dose + CT + PTV
Dataset-C	Dose + CT

Annotate:* CT* Computed tomography;* PTV* Planning target volume;* OARs* Organs at risk

### Model architecture

**Fig. 1:**
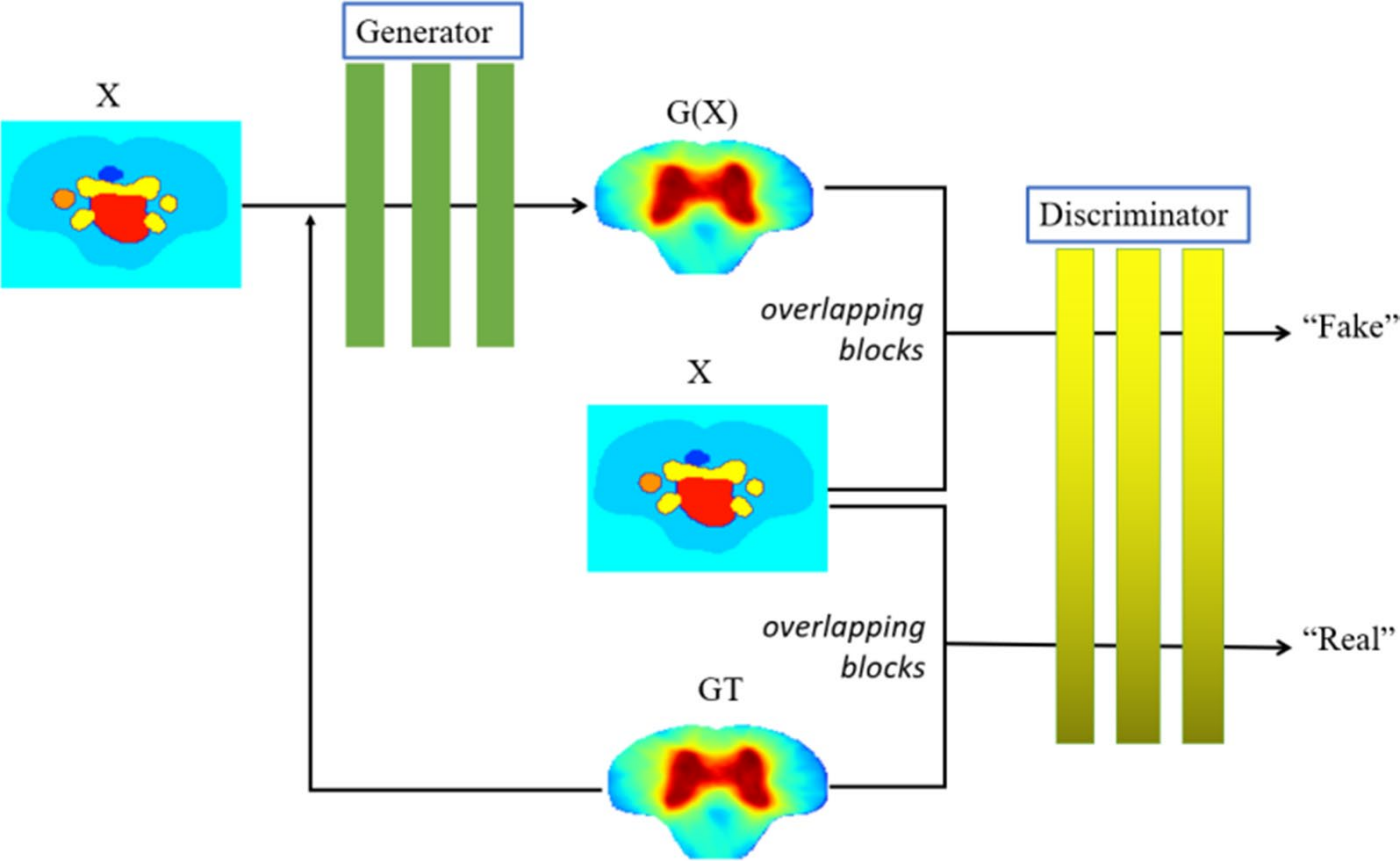
The overall framework and workflow of the generator and discriminator networks: X denotes the stack data of various input data; GT and G(X) denote the 3D clinical and predicted dose distribution respectively

With the focus of the establishment of correlation mapping relations between multichannel data consisting of anatomical features and medical images mentioned above with the radiation dose distribution at the voxel-voxel level, the network architecture adopted in this study mainly combined the two deep learning ideas of cGAN and full-scale fusion of feature information Encoding-Decoding structures. The overall structure of the model involved in this study which was presented as a 3D-cGAN-based framework, is shown in Fig. 1. The model was composed of a pair of discriminator and generator that confronted and promoted each other in the image generation process.

**Fig. 2:**
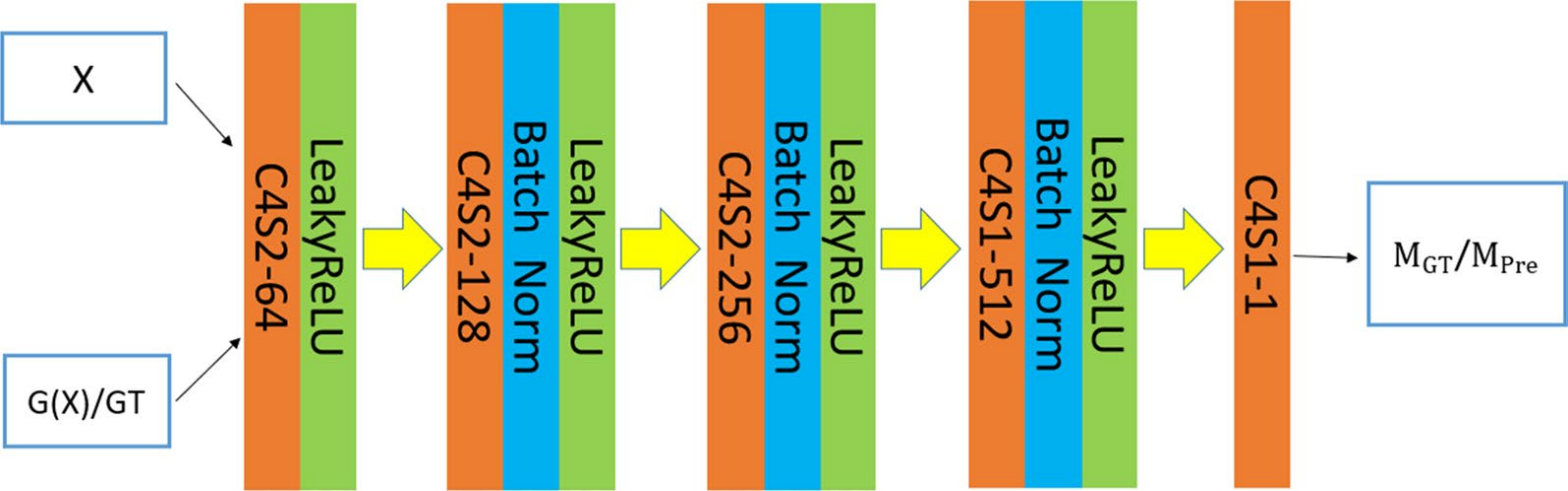
The architecture of the discriminator contains a total of 3 convolution layers with a stride of 2 and two convolution layers with a stride of 1, as well as the related normalization layer and activation layer. Each element in the 3D probability value matrixes has a 70*70*70 receptive field

The discriminator used in this study was a 3D-Patch-GAN classifier discerning locally overlapping image blocks instead of a traditional classifier based on the entire image. The convolutional neural network architecture of the discriminator is shown in Fig. 2. The input of the discriminator was a concatenated array of multichannel mask or CT image data and a dose distribution map matrix. Additionally, the synthetically predicted dose distribution or the clinical one were distinguished by identification probability values in [0,1]. Probability values corresponding to overlapping image blocks constituted the 3D probability value matrixes ${M}_{GT}$ for the ground truth and ${M}_{Pre}$ for the predicted dose map, and a combination of the binary cross entropy (BCE) and sigmoid function was adopted as the objective function of the discriminator :

1 $${L}_{D\_GT}=BCE\left\{\sigma \right(D\left(X|GT\right),1\}=-\frac{1}{n}\sum _{i=1}^{n}{log}\left(\frac{1}{1+{e}^{{-D\left(X\right|GT)}_{i}}}\right) , {D\left(X\right|GT)}_{i}\in {M}_{GT}$$

2 $${L}_{D\_Pre}=BCE\{\sigma \left(D\left(X|Pre\right),0\right\}=-\frac{1}{n}\sum _{i=1}^{n}{log}\left(1-\frac{1}{1+{e}^{{-D\left(X\right|Pre)}_{i}}}\right) , {D\left(X\right|Pre)}_{i}\in {M}_{Pre}$$

3 $${L}_{D}=\frac{{L}_{{D}_{GT}}+{L}_{{D}_{Pre}}}{2}$$

where X denotes the input data and ${L}_{D\_GT}$ or ${L}_{D\_Pre}$ denotes the loss items of the discriminator for the ground truth or prediction dose map, respectively. The discriminator’s output probability value $D\left(X\right|GT)$=1 represents the concatenated input data containing the clinical plan dose distribution map, while $D\left(X\right|Pre)$=0 represents the dose distribution from synthetic prediction, under ideal-trained conditions.

**Fig. 3:**
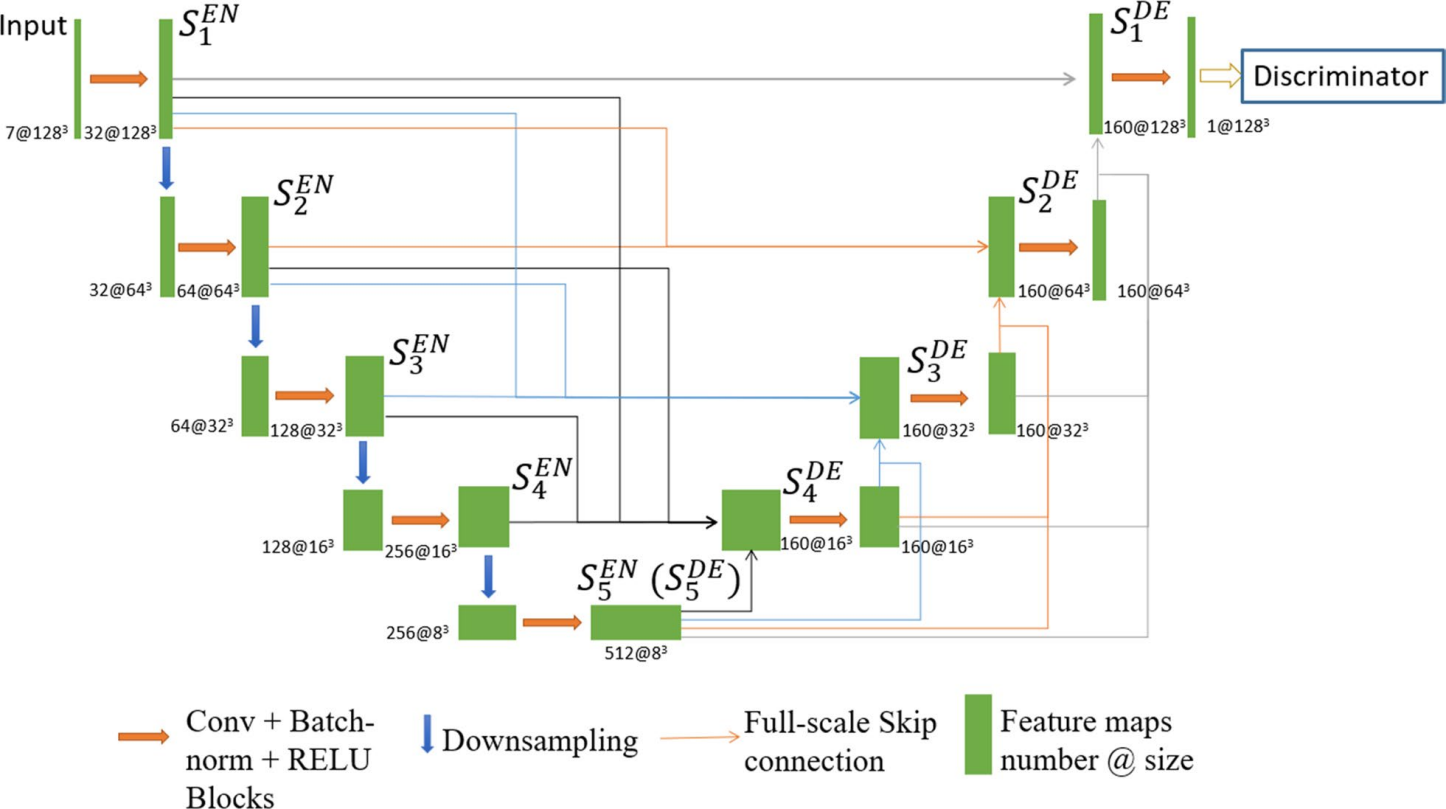
The proposed encoding-decoding generator architecture improved by UNet3+. ${S}_{i}^{EN}$and ${S}_{i}^{DE}$ represent the encoding and decoding steps respectively. The size and the number of channels are marked below the feature maps. The orange and blue arrows represent the convolution block and the downsampling blocks, and the straight lines with arrows in the figure represent the full-scale feature connection and fusion operation

As a full-scale feature connection deep learning network model, UNet3 + was originally proposed for medical image segmentation [33]. However, in this study, the novel framework was modified adaptively to carry out the dose distribution prediction based on the multichannel input and play the role of the generator of the cGAN model. The network architecture is shown in Fig. 3. ${S}_{i}^{EN}$and ${S}_{i}^{DE}$ represent steps of different levels of encoding and decoding, respectively, where a larger index i represents a higher level. At the beginning of the network, three convolutional layers with a convolution kernel size of 3*3*3 and stride of 1 were applied to extract image features, compared with a 7*7*7 convolution kernel, increasing the depth of the network to improve the nonlinear expression ability as well as to establish fewer parameters, which integrated 32 feature maps initially. In the subsequent encoding part of the network, the max-pooling layers preset were substituted by convolutional layers to avoid loss of feature information pertaining to location and intensity. The image was scaled to 1/8 of the original size as four downsamplings were performed during encoding. Between each two down-samplings, two convolution layers with a kernel size of 3*3*3 and stride of 1 were implemented. Furthermore, the number of feature maps was doubled, as conventional practice, each time downsampling was performed and the number of feature maps at each level of encoding step was 64, 128, 256, 512 respectively. As for decoding network, upsamplings instead of transposed convolutions were implemented in each step followed by convolution layers in order to prevent the checkerboard effect resulting from uneven overlap for the predicted dose distribution maps. The input of each step of the decoding network, apart from the decoding layer ${S}_{5}^{De}$, was associated with the output of the corresponding or low-level encoding and decoding steps to achieve the fusion of the full-scale features of delineation masks, CT images and clinical dose maps. The output of the encoding steps or the low-level decoding steps was upsampled or downsampled combined with the convolutional layers to keep the size of the feature maps consistent. Additionally, the number of multiscale feature maps to be concatenated was set to 32 equally. Since the encoding and decoding networks were all composed of 5 steps, 160 feature maps were separately fed to each decoding step. Considering the accuracy and stability of the generator for dose prediction, an objective function composed of three loss terms was proposed. First, for the purpose of joining the generator and the discriminator based on the overall structure of the cGAN, the adversarial loss term of the generator with the same form as the discriminator was adopted:

4 $${L}_{G\_cGAN}=BCE\left\{\sigma \right(D\left(X|G\left(X\right)\right),1\}=-\frac{1}{n}\sum _{i=1}^{n}{log}\left(\frac{1}{1+{e}^{{-D\left(X\right|G\left(X\right))}_{i}}}\right) , {D\left(X\right|G\left(X\right))}_{i}\in {M}_{G\left(X\right)}$$

where $G\left(X\right)$ denotes the dose map predicted by the generator based on the input delineation or CT data, $D\left(X|G\left(X\right)\right)$ denotes the identification probability value of the output result of the generator based on each overlapping image block by the discriminator, ${M}_{G\left(X\right)}$ represents a matrix constituted of probability values, and ${L}_{G\_CGAN}$ denotes the adversarial loss term of the generator. Second, the adjacent voxels difference loss term (AVD-loss) was adopted:

5 $${L}_{G\_AVD}=\sum _{i}^{{m}_{T}-1}\left\{{(({GT}_{i+1}-{GT}_{i})-({G\left(X\right)}_{i+1}-{G\left(X\right)}_{i}))}^{2}\right\}+\sum _{j}^{{m}_{C}-1}\left\{{(({GT}_{j+1}-{GT}_{j})-({G\left(X\right)}_{j+1}-{G\left(X\right)}_{j}))}^{2}\right\}+\sum _{k}^{{m}_{s}-1}\left\{{(({GT}_{k+1}-{GT}_{k})-({G\left(X\right)}_{k+1}-{G\left(X\right)}_{k}))}^{2}\right\}$$

where ${m}_{T}$, ${m}_{C}$, and${m}_{S}$ denote the numbers of transverse, coronal and sagittal slices of the 3D dose distribution image, respectively. ${GT}_{i}$, ${GT}_{j}$, and ${GT}_{k}$ denote the dose value matrixes of certain slices of the three sections above for the clinical dose distribution map, and ${G\left(X\right)}_{i}$, ${G\left(X\right)}_{j}$, and ${G\left(X\right)}_{k}$ denote the predicted dose distribution maps of the generator. ${L}_{G\_AVD}$ denotes the loss term which attempts to keep the edge features by minimizing the adjacent voxel dose value difference. The AVD-loss also removes the blur problem in the predicted dose, and maintains the sharpness of the image. Finally, the ${L}_{1}$ distance loss term was added to the optimization process of the generator:

6 $${L}_{G\_L1}={\left|\right|GT-G\left(X\right)\left|\right|}_{1}$$

where GT denotes the clinical dose, G(X) denotes the predicted dose, and ${L}_{G\_L1}$ denotes the ${L}_{1}$ loss term. Therefore, the total objective function of the generator can be obtained as:

7 $$L_{G} = \lambda _{{cGAN}} \cdot L_{{G\_cGAN}} + \lambda _{{G\_AVD}} \cdot L_{{G\_AVD}} + \lambda _{{L1}} \cdot L_{{G\_L1}}$$

where ${\lambda }_{cGAN}$, ${\lambda }_{G\_AVD}$ and ${\lambda }_{L1}$ denote the weighting parameters for balancing the adversarial loss term, the adjacent voxels dose difference loss term and the L1 loss term respectively.

The generator and the discriminator were trained by turns so that the capability of both could be mutually promoted in the adversarial process. For the discriminator, batch normalization and leaky rectified linear unit (Leaky ReLU) layers were adopted after each convolutional layer, while batch normalization and rectified linear unit (ReLU) layers were adopted for the generators. In particular, the hyperbolic tangent was used as activation function at the end of the generator. The total number of parameters of the involved generator and discriminator models were 11.077 M and 29.026 M respectively. For the whole deep learning network, Kaiming initialization was adopted. The Adam solver with high computational efficiency and stability was selected as an optimizer, in which the momentum parameters were set to ${\beta }_{1}=$0.5 and ${\beta }_{2}=$0.999. The initial learning rate of 0–200 epochs was set to 0.002, and attenuated linearly to 0 in 200–500 epochs. Two NVIDIA Tesla V100S GPUs were used for model training and testing in this study and the memory of each graphics card was 32GB. Considering to maximize the memory utilization of the graphics card, the batch size was set to 4. In addition, to increase the data sample and to prevent over-fitting, data augmentation was performed on the training set. Specifically, each input data was firstly randomly cropped as a 96*96*96 dose array and then randomly rotated by 90°, 180° or 270° in the transverse planes. The model training results of all epochs were recorded to research the impact of the training epoch on the model performance and to choose the optimal epoch to test.

### Performance analysis

Multiple methods were adopted to evaluate the proposed dose prediction methods. Based on this, the results obtained from the three different input datasets mentioned above were compared. First, not only the dose difference statistical histogram but also the mean absolute error (MAE), $MAE={\sum }_{i}^{m}\left|{GT}_{i}-{G\left(X\right)}_{i}\right|$, were calculated to compare the clinical and predicted dose distribution maps voxel by voxel, where *i* denotes the index of the voxel, and *m* denotes the total number of voxels. Second, the dose-volume histogram (DVH) curve was obtained to intuitively evaluate the consistency of the PTV and OARs dose distribution between the clinical and the dose maps predicted from three different datasets. Third, to specifically evaluate the clinical indicators of the clinical and predicted dose, the dosimetry indexes (DI) of the PTV or OARs were adopted, which included ${D}_{p}$ and ${V}_{d}$, where ${D}_{p}$ denotes the minimum dose received by volume in percentage of *p*, and ${V}_{d}$ denotes the maximum volume percentage receiving dose *d*. With respect to PTV, we calculated ${D}_{99\%}$, ${D}_{98\%}$, ${D}_{95\%}$, ${D}_{50\%}$, and ${D}_{2\%}$. In addition, we also used the homogeneity index (HI) and conformity index (CI):

8 $$HI=\frac{{D}_{2\%}-{D}_{98\%}}{{D}_{50\%}}$$

9 $$CI = \frac{{V_{{TP}} ^{2} }}{{V_{T} \cdot V_{P} }}$$

where ${V}_{T}$ denotes the volume of the PTV, ${V}_{P}$ represents the area covered by the prescribed dose, and ${V}_{TP}$ denotes the PTV area covered by the prescribed dose. Furthermore, ${D}_{max}$ and ${D}_{mean}$, which denote the maximum and average dose respectively, are taken into consideration for OARs. In addition, the ${V}_{40}$ of the bladder and rectum were considered. The paired-sample t-test was adopted, in which the threshold of statistical significance was set to P < 0.05, to statistically analyze the results of dose prediction. The Dice similarity coefficients (DSC) between the 3D isodose volumes of the clinical and predicted dose distribution images were calculated:

10 $$DSC\left(GT,Pre\right)=\frac{2*|{V}_{G{T}_{ISO} }\bigcap {V}_{Pr{e}_{ISO}}|}{\left|{V}_{G{T}_{ISO} }\right|+\left|{V}_{G{T}_{ISO} }\right|}$$

where ${V}_{G{T}_{ISO} }$ denotes the clinical isodose volume and ${V}_{Pr{e}_{ISO}}$ denotes the predicted isodose volume. Using the same deep learning architecture, Model-A, Model-B and Model-C were trained based on different input data of Dataset-A, Dataset-B and Dataset-C, respectively. In this study, all predicted dose distribution results based on three different models were compared with clinical dose maps in the same way. Finally, the method of gamma analysis was adopted to further evaluate the results of the dose distributions predicted by different models at the voxel level. The gamma passing rate (GPR) were calculated for PTV, OARs and the global, where the dose difference and distance-to-agreement (DTA) criteria were set to 3% and 3 mm respectively, with a dose threshold of 10% of the prescribed value. This facilitated us to simultaneously evaluate the location and dose value differences in 3D space.

## Results

**Fig. 4:**
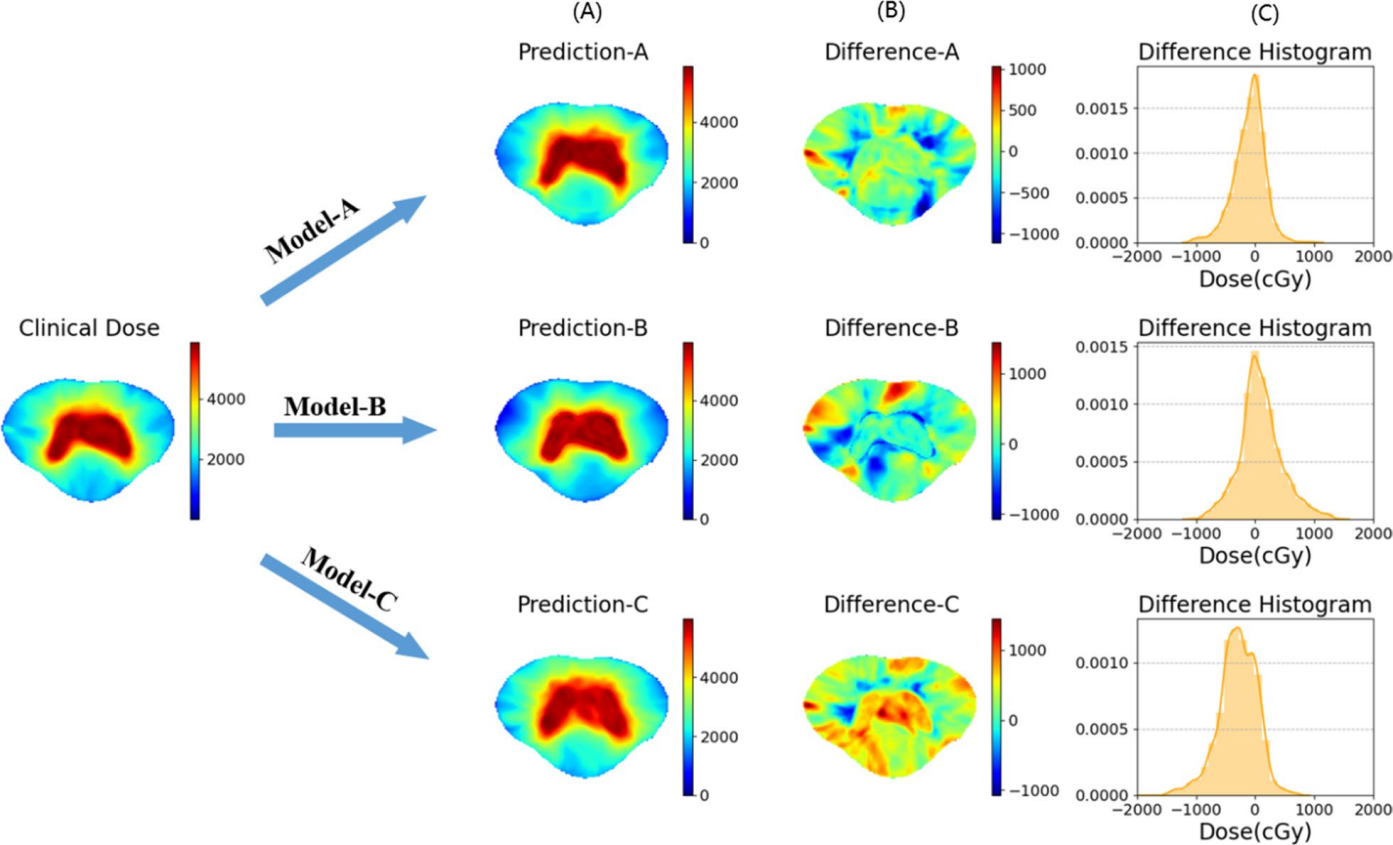
An example shows the difference between the predicted and clinical dose of cervical cancer in the transverse position, and the accuracy of the prediction is illustrated. The color bar is shown in units of cGy

The dose color wash for a test case is shown in Fig. 4. Difference distribution maps between prediction and clinical dose are provided. Visually, the predicted doses of the three different input datasets mentioned above are compared to the clinical dose. In addition, we computed a 1D histogram of difference distribution maps, which can reveal prediction bias and accuracy.

**Fig. 5:**
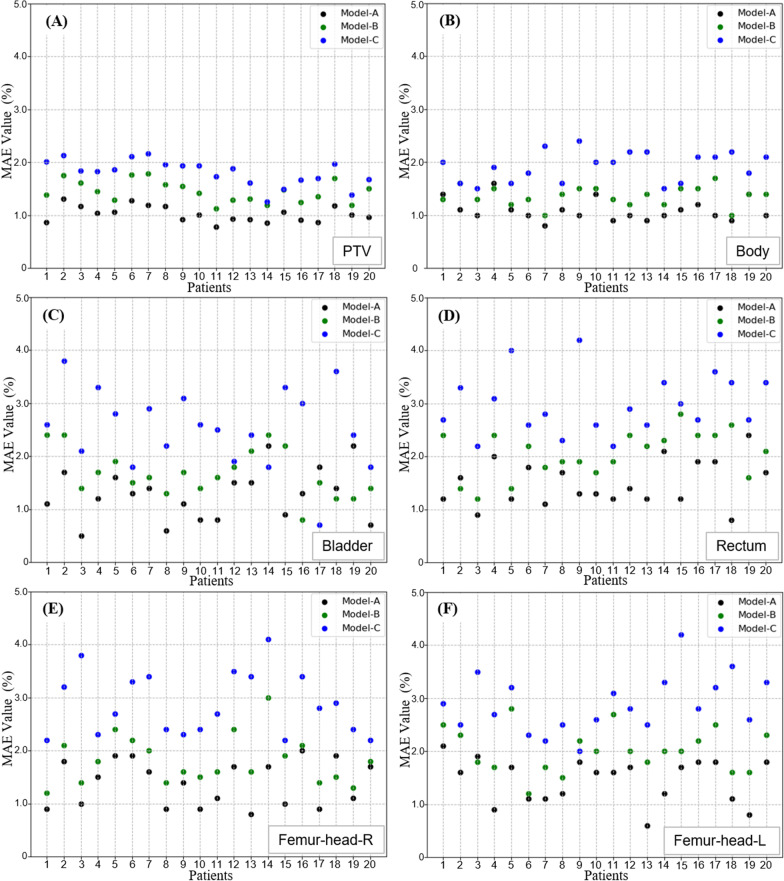
Mean absolute error (MAE) results of each test patient. The calculation areas include the whole body (**A**), PTV (**B**), bladder (**C**), rectum (**D**), right femoral head (**E**) and left femoral head (**F**)

The results of MAE on the testing set is shown in Fig. 5, which are calculated with contoured masks of the body, PTV and OARs. Model-A has the smallest average MAE values, which are 1.1 ± 0.2%, 0.8 ± 0.2%, 1.3 ± 0.5%, 1.5 ± 0.35%, 1.5 ± 0.4% and 1.4 ± 0.4% for the whole body, PTV ,bladder, rectum, right and left femoral heads, respectively; Model-B has the second highest MAE, which are 1.4 ± 0.2%, 1.2 ± 0.3%, 1.7 ± 0.4%, 2.1 ± 0.6, 2.0 ± 0.4% and1.8 ± 0.5%; Model-C has the highest MAE, which are1.9 ± 0.3% and 1.9 ± 0.5% for body and PTV, and the average values ranged from 2.6 ± 0.8% to 3.1 ± 0.6% for OARs. The MAE results of the body in the 7-fold cross-validation on the training-validation set are shown in Table 2. These results fully prove the reliability of our dose prediction model.

**Table 2:** The mean absolute error results of the body for different groups in seven fold cross-validation

Groups of cross-validation	Model-A		Model-B		Model-C	
	MAE-A (%)	*P*_*1*_	MAE-B (%)	*P*_*2*_	MAE-C (%)	*P*_*3*_
1	0.9 ± 0.4	0.102	1.3 ± 0.3	0.389	1.8 ± 0.3	0.221
2	1.1 ± 0.6	0.930	1.5 ± 0.3	0.405	2.2 ± 0.4	0.089
3	1.0 ± 0.3	0.814	1.2 ± 0.2	0.097	2.3 ± 0.4	0.080
4	1.0 ± 0.4	0.858	1.4 ± 0.3	0.762	2.0 ± 0.2	0.035
5	1.3 ± 0.4	0.791	1.5 ± 0.2	0.544	1.9 ± 0.6	0.216
6	1.4 ± 0.6	0.067	1.6 ± 0.6	0.085	2.3 ± 0.5	0.073
7	0.9 ± 0.2	0.083	1.3 ± 0.3	0.230	1.8 ± 0.3	0.229

Annotate: MAE-A, B, C represent the mean absolute error of each cross-validation group of Model-A, B, C, respectively. *P*_*1*_, *P*_*2*_, *P*_*3*_, represent the paired t-test results of each cross-validation group and the test group of Model-A, B, C, respectively

**Fig. 6:**
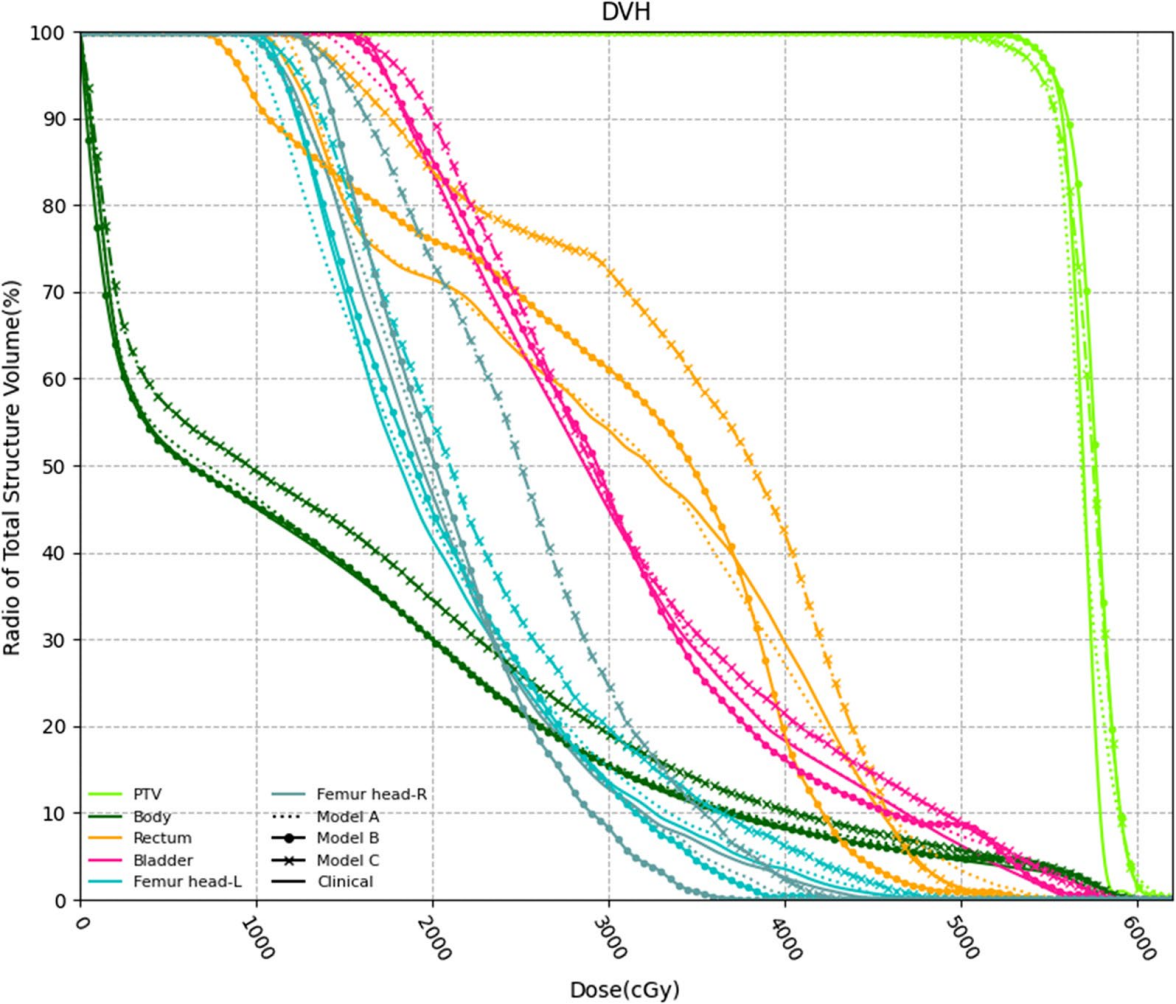
Comparison between the dose-volume histogram (DVH) of the clinical plan for a test case and the DVH based on the various models mentioned above

Figure 6 shows an example DVH comparison between the clinical dose and predicted dose from a test case. The solid line represents the clinical dose, while the dashed line represents the predicted dose from various dataset. As shown in Fig. 6, the predicted DVH is very close to the DVH derived from the dose distribution of the clinical radiation therapy plan, especially for Model-A and Model-B.

**Fig. 7:**
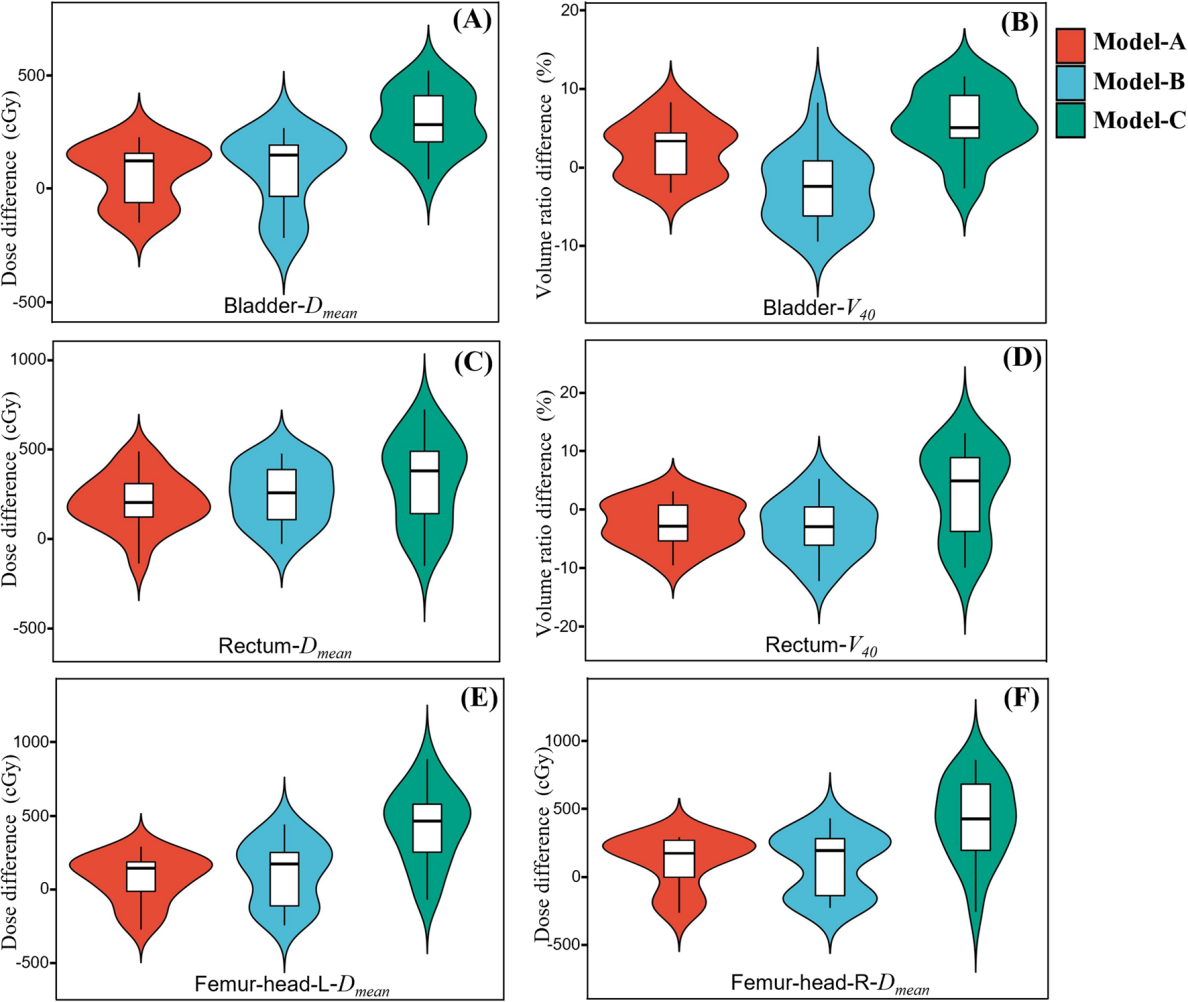
The difference of dosimetry indexes between the predicted and clinical dose for OARs, including the *D*_*mean*_ of the bladder (**A**), rectum (**C**), left femoral head (**E**) and right femoral head (**F**), and the *V*_*40*_ of the bladder (**B**) and rectum (**D**)

For the PTV, the prediction results of our proposed model are summarized in Table 3. Obviously, from a statistical point of view, for the D_99%_, D_98%_, and D_95%_ of the PTV of Model A and Model-B, there are no significant differences between the ground truth and predicted results. For all OARs, the D_max_ and D_mean_ dose prediction results are summarized in Table 4. Table 4 also includes the V_40_ dosimetry index for the bladder and rectum. There are also no significant differences between the D_mean_ prediction results of Model-A and Model-B and the ground truth. In general, the model we proposed predicts dosimetry indicators well. And the difference of the dosimetry indexes of OARs corresponding to the three models and ground truth dose were demonstrated in Fig. 7.

**Table 3:** The average dosimetry results of PTV for 20 test patients. Seven dosimetry indexs, ${D}_{99\%}$, ${D}_{98\%}$, ${D}_{95\%}$, ${D}_{50\%}$, ${D}_{2\%}$, HI and CI, are included

Dosimetry indexes												
Models	${\varvec{D}}_{90\varvec{\%}}$			${\varvec{D}}_{98\varvec{\%}}$			${\varvec{D}}_{95\varvec{\%}}$			${\varvec{D}}_{50\varvec{\%}}$		
	Predicted	Clinical	*P*-val	Predicted	Clinical	*P*-val	Predicted	Clinical	*P*-val	Predicted	Clinical	*P*-val
Model-A	53.4 ± 0.8	53.3 ± 0.6	**0.596**	54.2 ± 0.5	54.2 ± 0.4	**0.449**	55.1 ± 0.3	55.0 ± 0.3	**0.104**	57.0 ± 0.3	56.9 ± 4.0	**0.061**
Model-B	53.0 ± 0.9		**0.108**	54.0 ± 0.4		0.025	55.0 ± 0.3		**0.683**	57.3 ± 0.3		< 0.001
Model-C	50.6 ± 2.4		< 0.001	52.4 ± 1.7		< 0.001	54.3 ± 1.0		0.002	57.7 ± 0.3		< 0.001

Dosimetry indexes									
Models	${\varvec{D}}_{2\varvec{\%}}$			**HI**			**CI**		
	Predicted	Clinical	*P*-val	Predicted	Clinical	*P*-val	Predicted	Clinical	*P*-val
Model-A	59.6 ± 0.7	58.5 ± 03	< 0.001	0.1 ± 0.0	0.1 ± 0.0	**0.086**	0.8 ± 0.0	0.8 ± 0.0	**0.071**
Model-B	59.6 ± 0.5		< 0.001	54.2 ± 1.4		**0.073**	0.8 ± 0.0		**0.068**
Model-C	60.0 ± 0.4		< 0.001	54.2 ± 1.4		< 0.001	0.6 ± 0.0		< 0.001

The bold values denote that the difference of the dosimetry indexes between the predicted and clinical dose is not statistically significant

Annotate: ${D}_{p}$ denotes the minimum dose received by volume in percentage of *p*;* HI* Homogeneity index;* CI* Conformity index

**Table 4:** The average dosimetry results of OAR for 20 test patients. Three dosimetry indexs, ${D}_{max}$, ${D}_{mean}$, and ${V}_{40}$, are included

Dosimetry indexes										
OARS	Models	${\varvec{D}}_{\varvec{m}\varvec{a}\varvec{x}}$			${\varvec{D}}_{\varvec{m}\varvec{e}\varvec{a}\varvec{n}}$			${\varvec{V}}_{40}$		
		Predicted	Clinical	*P*-val	Predicted	Clinical	*P*-val	Predicted	Clinical	*P*-val
Rectum	Model-A	57.5 ± 4.1	56.5 ± 3.3	**0.262**	32.1 ± 4.9	32.2 ± 4.9	**0.283**	27.0 ± 14.2	27.6 ± 13.1	**0.276**
	Model-B	57.2 ± 3.0		**0.151**	32.3 ± 5.0		**0.357**	26.0 ± 13.5		**0.105**
	Model-C	57.6 ± 2.0		< 0.001	36.4 ± 4.2		< 0.001	40.0 ± 17.0		< 0.001
Bladder	Model-A	60.3 ± 1.2	58.7 ± 0.6	< 0.001	33.4 ± 3.1	33.5 ± 3.1	**0.157**	29.0 ± 10.0	28.7 ± 9.6	**0.093**
	Model-B	61.6 ± 2.0		< 0.001	33.6 ± 3.1		**1.126**	27.6 ± 9.6		**0.080**
	Model-C	60.1 ± 1.0		< 0.001	36.0 ± 2.8		< 0.001	34.0 ± 10.0		< 0.001
Femur-head-L	Model-A	42.0 ± 4.5	45.0 ± 5.7	< 0.001	21.4 ± 3.5	21.4 ± 3.5	**0.256**			
	Model-B	42.3 ± 7.1		**0.071**	21.3 ± 1.4		**0.083**			
	Model-C	49.2 ± 4.0		< 0.001	21.4 ± 3.5		< 0.001			
Femur-head-R	Model-A	43.0 ± 3.0	45.8 ± 4.1	< 0.001	24.7 ± 3.0	21.9 ± 2.6	**0.159**			
	Model-B	41.2 ± 4.8		**0.057**	21.9 ± 2.6		**0.136**			
	Model-C	49.2 ± 4.0		< 0.001	25.9 ± 2.2		< 0.001			
Body	Model-A	62.3 ± 1.8	60.1 ± 0.8	< 0.001	14.8 ± 1.9	14.8 ± 1.9	**0.569**			
	Model-B	62.7 ± 1.4		< 0.001	14.7 ± 2.0		**0.428**			
	Model-C	63.0 ± 0.9		< 0.001	16.0 ± 2.1		< 0.001			

The bold values denote that the difference of the dosimetry indexes between the predicted and the clinical dose is not statistically significant

Annotate: ${D}_{max}$ and ${D}_{mean}$ denote the maximum and mean dose for the organs; ${V}_{40}$ denotes the maximum volume percentage receiving dose 40 Gy

**Fig. 8:**
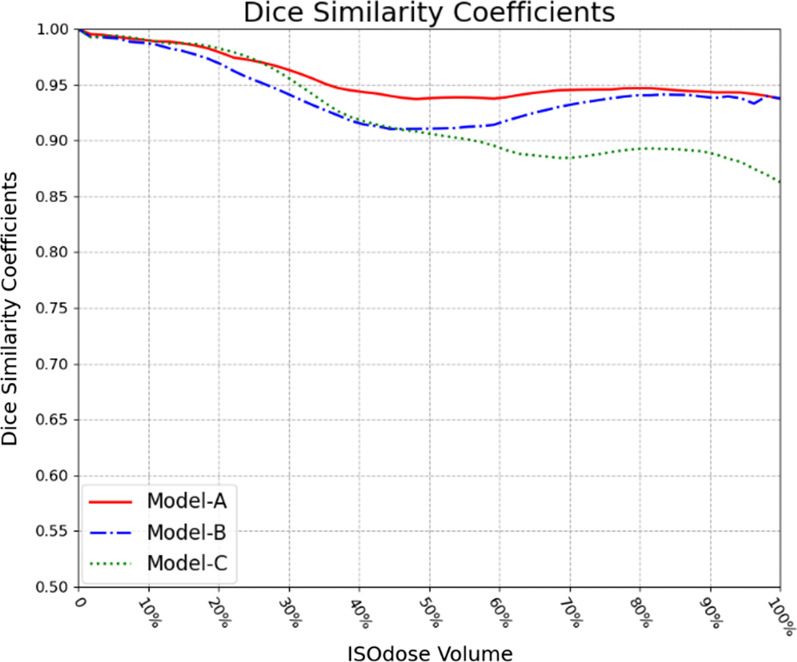
Dice similarity coefficients (DSCs) between clinical and predicted isodose volumes for test cohort

Figure 8 shows the average DSC results of the 20 test cohorts. The range of DSC value is 0–1, the best prediction result is 1 and the worst is 0 [42]. The average DSC for different isodose volumes of Model-A and Model-B is greater than 0.91, which represents an acceptable prediction result. Model-A reaches its peak, and the DSC is basically greater than 0.94. In the area where the isodose volume is less than 50%, the DSC of Model-C is acceptable, but after that, the DSC gradually decreases in the range of 0.86 to 0.91, and the difference between Model-A and Model-B gradually increases.

**Fig. 9:**
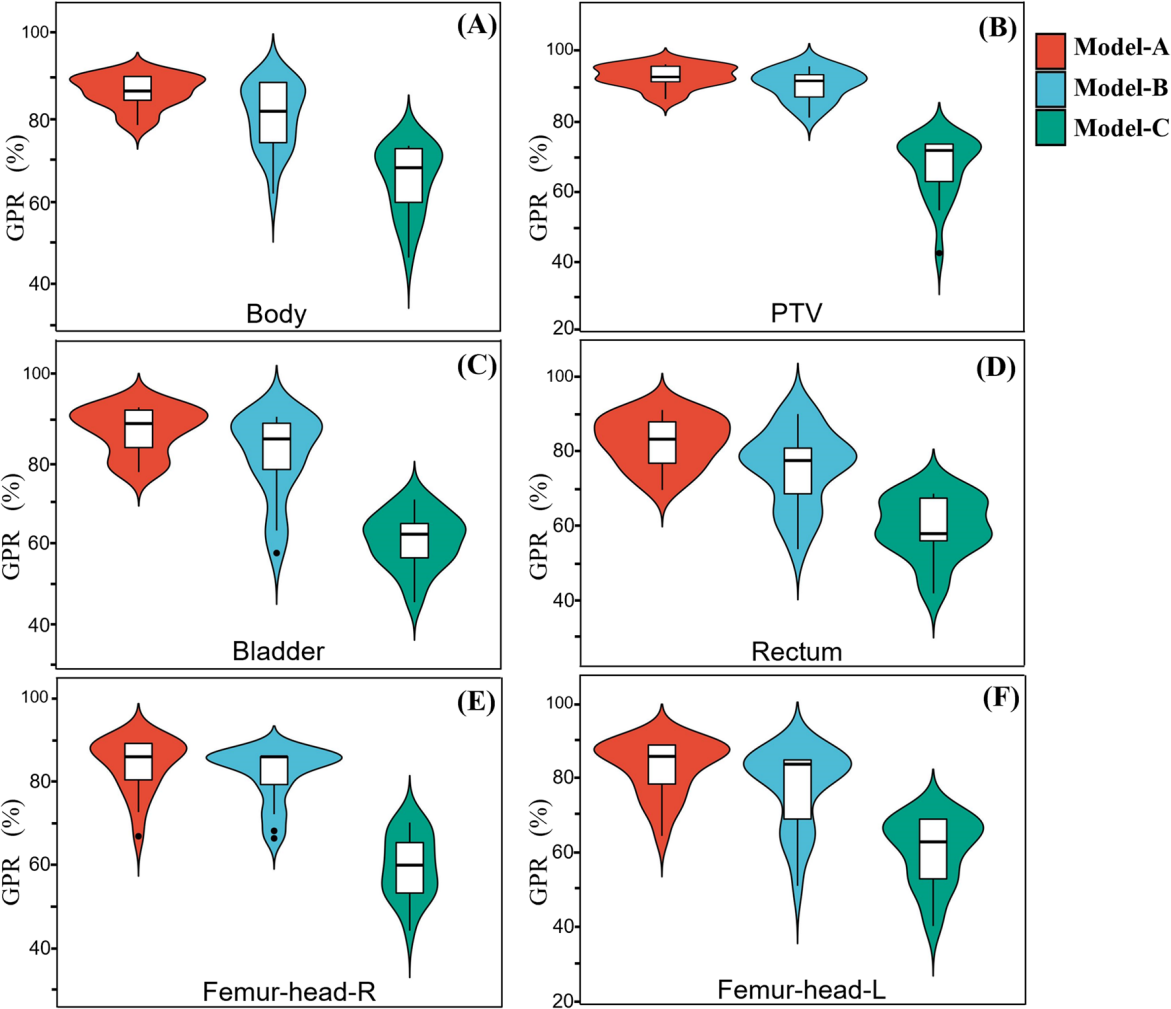
The gamma passing rates (GPR) of dose distribution predicted by different models for test cohort. The contoured structures include the whole body (**A**), PTV (**B**), bladder (**C**), rectum (**D**), right femoral head (**E**) and left femoral head (**F**)

For 20 test cases, the GPR of PTV, OARs and the whole body was calculated, as shown in Fig. 9. The GPR results (mean ± standard deviation) of the body were 90.1 ± 7.9%, 87.3 ± 9.3% and 66.4 ± 10.2 for Model-A, Model-B and Model-C, respectively. And for the PTV the GPR values were 95.2 ± 4.7%, 92.1 ± 6.9% and 68.5 ± 8.8%. For Model-A, the average GPR values of the four OARs were ranged from 81.4 to 86.7%, and the GPR of the bladder and femoral heads were all greater than 85%. For Model-B, the average GPR of OARs were ranged from 80.8 to 85.4%. Model-C performed the worst in the GPR evaluation of OARs, ranged from 60.7 to 63.4%.

## Discussion

In this study, we innovatively proposed a new full-scale feature fusion 3D-cGAN-based dose distribution prediction deep learning model. To our knowledge, we were the first to explore the influence of input data on three-dimensional dose distribution prediction in conjunction with a full-scale fusion and generative adversarial architecture, and in particular, we tried to use only CT images and the PTV as model input data and evaluated the feasibility. Owing to the similar target area structure, beam settings and uniform prescription standards, the dose distribution of the cervical cancer VMAT plan is highly consistent in the data of different patients. This makes the use of deep learning methods feasible for predicting the dose distribution of the cervical cancer VMAT plan. On the other hand, learning the contours of the OARs from CT images is theoretically supported by related segmentation studies [43–46]. Although in our research, the 3D-cGAN model was trained and made predictions in terms of cervical cancer cases treated with VMAT, this prediction method is also applicable to other treatment sites and techniques. The proposed deep learning method does not require manual extraction of any features. Instead, the model can automatically learn dose characteristics and associate voxels with dose distributions.

For the same network architecture, we explored three different multichannel input datasets, and the three models obtained showed different accuracy of dose distribution prediction. For the 20 tested patients, the MAE of the whole body of the three models was less than 2.2% relative to the prescribed dose of PTV, and less than 1.5% for Model-A and Model-B. Especially for Model-A, the mean value of the MAE was 1.1 ± 0.2%. The t-test results of multiple dosimetry indexes of the PTV and OARs of Model-A and Model-B also showed that there were no significant differences between clinical and expected results. In terms of the various evaluation indicators we considered, the input data of Model-C was the simplest and only included CT images, and its dose distribution prediction results were not as good as those of the other two models. However, Model-B can be comparable with Model-A. The prediction error between the two models is small in the high-dose region. Especially in the PTV structure, both are almost the same as the clinical dose. Model-C is very different in regard to the prediction of high-dose areas, which illustrates the importance of the PTV for high-dose area prediction. The input of Model-C contains only CT images, however, even if the PTV contours mainly reflect the information of the lesion, it undoubtedly includes the modification of the boundary made by the clinician based on the experience and the personal characteristics of the patient and the actual situation. There is a difference in the low-dose area of Model-A and Model-B. As shown in Fig. 8, for these regions, the DSC of Model-A is larger than that of Model-B. However, in terms of the evaluation indicators of OARs, the prediction results of the Model-B are not inferior to those of Model-A. This verifies the feasibility of dose prediction based only on CT images and PTV target areas. In addition to the PTV, the predictive model can learn the contour information of the OARs during the training process.

The results of DIs indicate that the proposed Model-A and Model-B predict better for dose values close to the prescription and average dose than the low-dose area and maximum value. Such as the D_2%_ indexes of PTV are significantly different from the predicted and clinical dose for both models, which may be related to its limited contribution to the loss function; For *D*_*99*_, *D*_*95*_ of PTV and *D*_*mean*_ of OARs, both of the models have no significant differences from the clinical, but the models performs poorly for *D*_*max*_. Such as for bladder, the shape and volume is related to the state of urine holding and some voxels overlap with PTV, which makes the prediction of that more complex and difficult than other OARs. A potential method is to localize or specify the global loss terms (L1 and AVD), such as to set adaptive weights for different masks or regions of interest. Using other normalization methods (such as instance or group-norm) may also works, but the impact of this on other evaluation indicators remains to be studied. The performance of the two models on *D*_*max*_ in OARs and body is inconsistent. For the rectum, the bladder and the body, the two models are comparable (Model-A is better on average values). However, Model-B performs better for the femoral heads than Model-A. This may due to the closer distance to high-dose area as well as the no overlapping with PTV, and the relaxed constraints of high dose during feature fusion in Model-B without masks of the femoral heads. The influences of the regional distance and input masks on *D*_*max*_ indexes will be further researched in our future work.

Recently, there are related studies for head and neck, breast, abdomen or pelvis, in which the treatment techniques include 3D-CRT, IMRT, VMAT and PBS, and the U-Net is basically adopted as architecture [47–50]. The evaluation indicators for performance analysis are similar in almost all related works and Ahn et al. used gamma analysis as a metric [47]. Our models use cGAN as the basic architecture, which exporting satisfactory prediction results. In fact, for the generator, we have also tried to use networks of U-Net or residual blocks, but the former has checkerboard effects in the predicted maps, and the latter, with identity mapping, causes the ${L}_{G\_L1}$ and ${L}_{G\_AVD}$ to converge too faster than ${L}_{G\_cGAN}$, which might lead to overfitting. Current related work mostly uses CT and delineated contours as input data, but we tried to remove OARs from the input data to improve the efficiency of prediction and the results show a certain feasibility. Compared with the existing research, the results predicted by the full-scale feature fusion 3D-GAN-based Model-A and Model-B we proposed are closer to the clinical dose distribution. Tomohiro K et al. used machine learning methods to predict 3D dose, and the MAE of the reported prostate cases was 5% [51]. Our prediction results of Model-A and Model-B show that the MAE values of the prediction error for the whole body are less than 1.4%. And we further calculate PTV and MAE of each OAR to reflect dose difference more specifically in 3D space. The results show that for the three models, the overall and local performance have the same trend, that is, the performance of Model-A, Model-B and Model-C decreases successively, and the performance of Model-A and Model-B is significantly better than that of Model-C. Liu Z et al. used ResNet-UNet as an IMRT dose prediction model in the case of nasopharyngeal carcinoma (NPC), and there was no statistical difference between the predicted result and clinical dose regarding the indicator of the average D_mean_ of the OARs [52]. Our forecast results show comparable accuracy. Our results for Model-A and Model-B show that the average DSCs of the test set are greater than 0.94 and 0.91, respectively. The DSC of Model-A is higher than the results of Nguyen D et al. (0.90), where ResNet-UNet was used to predict pelvic dose [31]. Model-B has a similar DSC value. In the evaluation of GPR, Model-A and Model-B both show superiority compared with other similar works, such as 5 mm/3% 3D GPR values ranged from 81 to 90% for PTV and OARs in the studies of Zhou et al. [53]. The GPR results further demonstrate that the 3D-cGAN-based model is suitable for dose distribution prediction tasks based only on CT image and PTV. The results of Model-C also demonstrate the limitation of the input data containing only CT images as for our model. In brief, our results are similar to the prediction accuracy of the above studies, and some dose indicators are better. In fact, integrating new mechanisms for the model in future work may further improve the prediction accuracy of Model-B and make Model-C feasible. Li et al. tried to achieve both segmentation and dose prediction based on CT images only, with the help of a multi-task attention adversarial network, where the introduction of the attention mechanism achieved interpretable evaluation to a certain extent while optimizing the feature fusion process [54].

The dose prediction model proposed can be used as a clinical tool to guide doctors and radiation physicists to ensure the quality of treatment plans. The proposed method of inputting only CT images and the PTV as the model further reduces the time cost compared with that of traditional dose prediction research. Clinicians and radiation physicists can use the proposed dose prediction model to immediately obtain the 3D dose distribution of the VMAT plan for cervical cancer patients after the PTV is delineated. On the one hand, clinicians can directly view the predicted 3D dose distribution and then compare it with other treatment plans to choose the most suitable treatment plan for the patient. On the other hand, physicists can improve the plan optimization strategy based on the predicted dose distribution, thereby reducing the inconsistency of the plan quality of different physicists. Finally, a clinically acceptable plan can be quickly designed, which can reduce the total planning time and improve the quality of the patient’s prognosis.

This study has some limitations. An obvious limitation is the data size. Although the full-scale connection and the 3D-cGAN-based structure can fully learn the correlation information between the voxels and dose distribution, collecting more data can further narrow the generalization gap and improve the prediction effect. Another limitation is that this study used only the bladder, rectum, and femur heads for evaluation. More detailed classification of human organs is one of the ways to improve the prediction effect. In addition, we try to use only medical image data and PTV as input data, without including OAR contours, but multimodal images, such as magnetic resonance imaging (MRI) images, are referenced during the delineation process in some cases, so adding multimodal images to the multichannel input data may make it easier for the network to extract the correct OAR contour information, thereby improving the accuracy of dose prediction. In fact, MRI of simulated positioning was initially considered as one of the input data channels, but we did not find enough cases with that especially considering of factors such as the quality of the delivery plans. There are two reasons leading to this. First, unlike 3D-CRT and IMRT, VMAT is a technology that is widely used in recent years. Second, under the premise of CT simulation positioning, radiotherapy physicians usually consider the financial burden of cancer patients. But the feasibility of dose prediction based only on medical images data and PTV contours, has been confirmed by our works.

In the future, this research will be further enriched, and the following methods will be used for improvement. First, we will apply our method to the radiotherapy dose distribution predictions of different treatment site and treatment technologies to verify the universality of the proposed methods. Second, considering that MRI is better than CT for imaging the bladder and other tissues, we will collect MRI images as input for the model in the future. Strategies can be mainly adopted for this work. The cervical cancer patients with MRI simulation positioning can be prospectively enrolled to collect enough cases. And it is possible to consider the research of other parts, such as cases of brain and breast patients, in which MRI simulation positioning is necessary. We hope that adding this type of information will significantly improve the performance of deep learning models and quantify its impact on dose prediction. Third, we will combine automatic delineation of OARs with dose prediction for automatic radiotherapy plan, both have their respective roles in planning and evaluation to balance the time cost and accuracy. The dose maps predicted without OARs can provide tremendous support and clinical application feasibility for such work. Based on deep learning networks and related optimization algorithms, such as optimization model with voxel dose constraints or target setting assisted by of predicted DVH, automatic TPS will be developed, ultimately serving clinicians and radiation physicists.

## Conclusion

We proposed a full-scale feature fusion 3D-cGAN-based cervical cancer VMAT plan dose prediction framework, and we researched and compared the influence of different input data compositions on 3D dose distribution prediction in conjunction with full-scale and generative adversarial architecture. In particular, we confirmed the feasibility of dose prediction based only on CT images and the PTV, without delineated contours of the OARs. The method proposed in this paper provides a simpler and more effective method for clinical dose assessment, radiotherapy planning assistance and automatic planning. With further improvement in our models and applications in the future, this method may play an important role in clinical work.

## Data Availability

The datasets used during the current study are available from the corresponding author on reasonable request.
